# Marijuana use and its correlates among school-going Jamaican adolescents: a finding from a national survey

**DOI:** 10.3389/fpsyt.2023.1324869

**Published:** 2024-01-05

**Authors:** Omid Dadras

**Affiliations:** ^1^Department of Global Public Health and Primary Care, University of Bergen, Bergen, Norway; ^2^Bergen Addiction Research, Department of Addiction Medicine, Haukland University Hospital, Bergen, Norway

**Keywords:** cannabis, marijuana, Jamaica, adolescents, substance use, risky behaviors

## Abstract

**Introduction:**

The recent data indicate almost a fifth of Jamaican adolescents used marijuana in the past 30 days. To ensure the optimal allocation of resources, a country-specific understanding of factors associated with marijuana use among adolescents is essential. Therefore, this study aimed to address this gap among adolescents aged 13–17 years in Jamaica.

**Methods:**

We analyzed data from the recent Jamaica Global School-Based Student Health Survey conducted in 2017. The sample consists of school-going Jamaican adolescents of 7th−12th grades. The prevalence of recent marijuana use was assessed and compared across different demographics, substance use, and risk behaviors using bivariate and multivariable logistic regression analyses.

**Results:**

Older adolescents and men had a higher likelihood of recent marijuana use. Psychosocial risks, such as loneliness, frequent worry, suicidal ideation, physical attacks, and school absenteeism, were associated with higher marijuana usage. Parental smoking increased the odds, whereas strong parental support and awareness decreased it. Other substance uses, especially amphetamine and tobacco products, had strong associations with marijuana use. Early initiation of substances was associated with a higher risk of marijuana use. Sexually active adolescents, especially those initiated before the age of 14 years, had higher rates of marijuana use.

**Conclusion:**

The intricate link between harmful and supportive psychosomatic and risk behaviors with recent marijuana use highlights the importance of holistic interventions and policies focusing on emotional health, parental guidance, substance education, and sexual activity implications.

## Introduction

Cannabis, commonly known as marijuana, holds a prominent position in the cultural and socioeconomic fabric of Jamaica. Historically rooted in the practices of the Rastafarian movement, its use has expanded beyond religious rituals to become a part of recreational activities among various age groups, particularly adolescents (1). With the increasing discussions surrounding the potential benefits and harms of cannabis use, understanding its prevalence and the factors associated with its consumption becomes particularly critical. Adolescence, marked by the age group of 13–17 years, is a crucial developmental phase characterized by physical, psychological, and social changes (2). During this period, the propensity for risky behaviors, including substance use, tends to rise due to a combination of curiosity, peer pressure, and brain development processes (3).

The Global School-based Student Health Survey (GSHS), a collaborative surveillance project designed to help countries to measure and assess behavioral risk factors and protective factors among students, has been pivotal in shedding light on such behaviors in various countries, including Jamaica (4). Given unique sociocultural context of Jamaica and its historically complex relationship with cannabis, understanding the landscape of cannabis use among its adolescents is essential [3]. The 2017 GSHS report reveals that nearly one in five Jamaican adolescents aged 13–17 years has used marijuana minimum once in their lives. This prevalence is more pronounced among older adolescents and is almost twice as high among men compared with their female counterparts (5).

To ensure the optimal allocation of resources for prevention and treatment, a country-specific understanding of the correlated demographic, substance use, and risk behaviors with marijuana use among adolescents is essential. Therefore, this study aimed to address the correlates of cannabis use among adolescents aged 13–17 years in Jamaica using secondary data from the GSHS 2017. The findings could also provide policymakers, educators, and health professionals with insights into the local dynamics of cannabis consumption, aiding in the formulation of targeted interventions and policies. By shedding light on this critical issue, the study aimed to contribute to the broader dialogue surrounding adolescent health and wellbeing in the Caribbean region.

## Methods

### Study setting

This study entails secondary data analysis using data from the 2017 Jamaica Global School-based Student Health Survey (GSHS). The Jamaica Global School-based Student Health Survey (GSHS) is a nationally representative sample survey of students in secondary schools. It is conducted in collaboration with the World Health Organization (WHO) and the Centers for Disease Control and Prevention (CDC). The survey focuses on various health-related behaviors and protective factors among students, such as dietary behaviors, hygiene, physical activity, mental health, and the use of substances such as tobacco and alcohol.

### Participants

The 2017 Jamaica Global School-Based Student Health Survey employed a two-stage cluster sample design. This method was chosen to yield data that would be representative of all students of grades 7th−12th in Jamaica. The use of a two-stage cluster sampling design ensures that the sample accurately reflects the characteristics of the target population, i.e., students in the specified grades in Jamaica. The school response rate was 84%, the student response rate was 71%, and the overall response rate was 60%. A weighting factor was applied to each student record to adjust for non-response and varying selection probabilities, and more details about the weighting factor calculation are provided in the GSHS Data User's Guide (6).

### Data collection

During a regular class session, students were given the standard GSHS questionnaire, including questions about demographics (age, sex, and grade), anthropometric characteristics (weight and height), dietary habits, personal and oral hygiene, mental health, violence and unintentional injuries, bullying, substance use (tobacco, alcohol, and illegal drugs), sexual and reproductive health, parental support, and physical activity. Before conducting the survey, students were briefed on its contents, ensured their confidentiality, and made aware that their participation was entirely voluntary.

### Outcome variables

Recent marijuana use was assessed by the question, “During the past 30 days, how many times have you used marijuana (also called ganja or weed)?,” and the answers were coded as binary variable (yes = 1; no = 0).

### Explanatory variables

#### Demographic variables

Age (11–18 years), sex (male or female), and grades (7–12) are the demographic variables.

#### Psychosocial risks and protective factors

Felt lonely was assessed by the question, “During the past 12 months, how often have you felt lonely?,” and the answers were coded as 1 (never/rarely), 2 (sometimes), 3 (most of the time), and 4 (always). Felt worried was assessed by the question, “During the past 12 months, how often have you been so worried about something that you could not sleep at night?,” and the answers were coded as 1 (no), 2 (1 time), and 3 (≥ 2 times). Suicide ideation was assessed by the question, “During the past 12 months, have you ever seriously considered attempting suicide?,” and the answers were coded as binary variable (yes = 1; no = 0). Suicide attempt was assessed by the question, “During the past 12 months, how many times did you actually attempt suicide?,” and the answers were coded as binary variable (yes = 1; no = 0). Physically attacked was assessed by the question, “During the past 12 months, how many times were you physically attacked?,” and the answers were coded as 0 (no) and 1 (yes). Bullying history was assessed by the question, ‘During the past 30 days, how many days were you bullied?', and the answers were coded as binary variable (yes = 1; no = 0). Missed school was assessed by the question, “During the past 30 days, how many days did you miss classes or school without permission?,” and the answers were coded as 1 (no), 2 (1–2 days), and 3 (≥ 3 days). Parental smoking was assessed by the question, “Which of your parents or guardians use any form of tobacco?,” and the answers were coded as binary variable (yes = 1; no = 0). Parental support was assessed by the question, “During the past 30 days, how often did your parents or guardians understand your problems and worries?,” and the answers were coded as 1 (never/rarely), 2 (sometimes), 3 (most of the time), and 4 (always). Parental awareness was assessed by the question, “During the past 30 days, how often did your parents or guardians really know what you were doing with your free time?,” and the answers were coded as 1 (never/rarely), 2 (sometimes), 3 (most of the time), and 4 (always).

#### Substance use variables

Ever amphetamine use was coded as 0 (no) and 1 (yes). Age at drug/cigarette/alcohol, as previous studies emphasized ages <14 as a time of heightened vulnerability for mental issues (7, 8), this variable was coded as 0 (<14) and 1 (≥14). The current use of cigarette/other tobacco products/alcohol drinking was coded as 0 (no) and 1 (yes). Got drunk/troubled drunk was assessed by the questions, “During your life, how many times did you drink so much alcohol that you were really drunk?” and “During your life, how many times have you got into trouble with your family or friends, missed school, or got into fights, as a result of drinking alcohol?,” and the answers were coded as 0 (no) and 1 (yes).

#### Risky sex behaviors

Ever had sex was coded as 0 (no) and 1 (yes). Age at first sex was coded 0 (<14) and 1 (≥14). Sexual partnership was assessed by the question, “During your life, with how many people have you had sexual intercourse?,” and the answers were coded as binary variable (yes = 1; no = 0). Condom use in the last sex was also coded as 0 (no) and 1 (yes).

### Statistical analysis

The descriptive statistics were employed to describe the distribution of sample demography, psychosocial risks and protective factors, substance use, and risky sex behaviors, and corresponding prevalence of recent marijuana use among Jamaican adolescents of grades 7–12. Logistic regression analysis was used to examine the likelihood of recent marijuana use across the explanatory variables, accounting for age and sex, and the results were reported as adjusted odds ratios (AOR) and 95% confidence intervals (95% CIs). This approach allowed for the estimation of the independent effects of the study variables on recent marijuana use, regardless of the potential confounding effects of age and sex (9), therefore allowing for targeted school-based interventions and preventive strategies for all students in grades 7–12 while providing valuable policy insight (10). Sampling design and weights were applied by defining the survey strata, primary sampling unit, and weight using STATA 17. The statistical significance level was set at *p* < 0.05.

## Results

### The associated demographic factors with marijuana use

Table 1 provides an overview of the characteristics and their association with recent marijuana use. Adolescents who were older than 14 years had a prevalence rate of 15.06% for marijuana use, which was significantly higher than their counterparts (8.00%). The odds of using marijuana for those older than 14 years were more than twice as high as for those aged 14 years and younger. Male adolescents exhibited a prevalence rate (16.78%) of marijuana use compared with female counterparts (9.18%). The odds of men using marijuana were double that of women.

**Table 1:** Demographic characteristics and their association with marijuana use among Jamaican adolescents in 7th−12th grades.

**Variable**	**Recent marijuana use**		
	**N (weighted %)**	**N (weighted %)**	**OR (95%CI)**
**Age group**			
≤ 14	628 (31.36)	49 (8.00)	Ref
>14	1,031 (68.64)	146 (15.06)	2.04 (1.21–3.42)^*^
**Sex**			
Male	755 (49.15)	111 (16.78)	2.00 (1.48–2.69)^*^
Female	900 (50.85)	83 (9.18)	Ref
**Grade**			
7th	41 (2.11)	3 (9.18)	Ref
8th	412 (22.07)	33 (8.31)	0.90 (0.28–2.88)
9th	557 (25.34)	66 (13.57)	1.55 (0.59–4.09)
10th	388 (23.99)	67 (17.64)	2.12 (0.88–5.12)
11th	208 (21.97)	22 (13.02)	1.47 (0.54–4.07)
12th	44 (4.51)	3 (6.34)	0.67

* *p*-value < 0.05.

### The associated psychosocial risks and protective factors with marijuana use

Table 2 presents the association of psychosocial risks and protective factors with recent marijuana use. Adolescents who reported always feeling lonely had more than two-fold higher odds of marijuana use as compared with those who never or rarely felt lonely. Adolescents feeling worried most of the time were more likely to report marijuana use in comparison with their counterparts who never or rarely felt worried. The odds of recent marijuana use were approximately 2 and 2.5 times higher among adolescents with suicidal ideation and attempt, respectively, as compared with their counterparts. Adolescents who had been physically attacked in the last 12 months had 83% increased odds of using marijuana when compared with those who had not been attacked. Adolescents who missed school for 3 or more days in the past month were almost three times more likely to use marijuana in comparison with those who did not miss school. Adolescents with parents who smoked had more than 2-fold higher odds of using marijuana as opposed to those whose parents did not smoke. Notably, increased parental support and awareness were associated with lower odds of marijuana use. Adolescents who reported always having parental support were 46% less likely to use marijuana. Additionally, those who always experienced parental awareness had 70% lower odds of using marijuana.

**Table 2:** Psychosocial risks and protective factors and their association with marijuana use among Jamaican adolescents in 7th−12th grades.

**Variable**	**Recent marijuana use**		
	**N (weighted %)**	**N (weighted %)**	**AOR (95%CI)**
**Felt lonely (last 12 months)**			
Never/rarely	837 (51.79)	97 (12.55)	Ref
Sometimes	500 (29.57)	51 (11.30)	0.95 (0.62–1.44)
Most of the time	208 (12.16)	19 (9.95)	0.89 (0.50–1.59)
Always	110 (6.48)	25 (22.98)	2.22 (1.27–3.86)^*^
**Felt worried (last 12 months)**			
Never/rarely	1,032 (62.79)	109 (11.45)	Ref
Sometimes	398 (24.31)	47 (13.53)	1.27 (0.69–2.33)
Most of the time	146 (8.79)	25 (18.24)	1.97 (1.05–3.72)^*^
Always	70 (4.10)	10 (15.8)	1.75 (0.76–4.04)
**Suicidal ideation**			
No	1,178 (75.06)	121(11.08)	Ref
Yes	422 (24.94)	62 (16.48)	1.94 (1.35–2.79)^*^
**Suicidal attempt**			
No	1,322 (82.08)	135 (11.20)	Ref
Yes	314 (17.92)	61 (21.78)	2.51 (1.66–3.80)^*^
**Physically attacked (last 12 months)**			
No	1,207 (73.44)	116 (10.41)	Ref
Yes	448 (26.56)	79 (19.66)	1.83 (1.23–2.73)^*^
**Bullying history (last 30 days)**			
No	1,180 (76.11)	124 (11.69)	Ref
Yes	395 (23.89)	60 (15.49)	1.37 (0.94–2.04)
**Missed school (last 30 days)**			
No	1,192 (71.43)	108 (9.75)	Ref
1–2 days	254 (16.49)	42 (15.98)	1.50 (0.87–2.59)
≥3 days	183 (12.09)	44 (26.52)	3.10 (1.69–5.68)^*^
**Parental smoking**			
No	1,056 (75.61)	93 (9.31)	Ref
Yes	363 (24.39)	63 (19.79)	2.38 (1.52–3.72)^*^
**Parental support**			
Never/rarely	759 (46.87)	107 (15.18)	Ref
Sometimes	354 (22.46)	37 (11.18)	0.64 (0.36–1.15)
Most of the time	193 (11.89)	20 (11.15)	0.64 (0.38–1.08)
Always	316 (18.78)	27 (9.13)	0.54 (0.29–1.00)^*^
**Parental awareness**			
Never/rarely	591 (37.79)	95 (17.99)	Ref
Sometimes	381 (22.76)	47 (13.25)	0.74 (0.54–1.01)
Most of the time	261 (16.12)	28 (10.61)	0.58 (0.36–0.95)^*^
Always	388 (23.33)	21 (5.64)	0.30 (0.17–0.53)^*^

* *p*-value < 0.05.

### The associated substance use with marijuana use

Table 3 presents the association between recent marijuana use and various substance use. A significant association was observed between amphetamine use and recent marijuana use. Specifically, those who use amphetamine were approximately 25 times more likely to use marijuana use compared with those who did not use amphetamine. Individuals currently smoking cigarettes were 13 times more likely to use marijuana, while those using other tobacco products were 14 times more likely, compared to peers who did not smoked cigarettes or used any other tobacco products. Additionally, those who started smoking cigarettes before the age of 14 years were twice as likely to use marijuana compared with those who initiated after 14 years. Alcohol use was associated with more than five times higher odds of recent marijuana use. Additionally, those with a history of getting drunk and troubling drunk were three to five times more likely to use marijuana as compared with those who had not, indicating a pronounced association with recent marijuana use. Adolescents who had their first drink before 14 years old also had approximately 3-fold higher odds of marijuana use than their counterparts.

**Table 3:** Substance use disorders and their association with marijuana use among Jamaican adolescents in 7th−12th grades.

**Variable**	**Recent marijuana use**		
	**N (weighted %)**	**N (weighted %)**	**AOR (95%CI)**
**Ever amphetamine use**			
No	1,534 (97.02)	155 (10.87)	Ref
Yes	46 (2.98)	35 (7.69)	24.49 (8.63–69.49)^*^
**Age at drug initiation (years)** ^a^			
< 14	140 (67.32)	132 (94.06)	0.46 (0.15–1.46)
≥14	64 (32.68)	62 (98.11)	Ref
**Current cigarette use**			
No	1,397 (85.29)	91 (6.83)	Ref
Yes	237 (14.71)	96 (49.77)	12.95 (8.17–20.52)^*^
**Current use of other tobacco products**			
No	1,458 (88.77)	109 (7.86)	Ref
Yes	179 (11.23)	85 (56.90)	14.29 (7.95–25.70)^*^
**Age at cigarette initiation (years)** ^b^			
< 14	62 (27.51)	72 (54.55)	2.14 (1.24–3.70)^*^
≥14	172 (72.49)	22 (37.61)	Ref
**Currently, drink alcohol**			
No	837 (51.43)	37 (4.66)	Ref
Yes	754 (48.57)	150 (22.22)	5.19 (1.24–3.70)^*^
**History of drunk (lifetime)**			
No	1,157 (68.25)	85 (8.01)	Ref
Yes	510 (31.75)	113 (25.14)	3.33 (2.22–5.00)^*^
**Troubling drunk**			
No	1,419 (84.79)	131 (9.56)	Ref
Yes	248 (15.21)	67 (34.32)	4.64 (2.55–8.47)^*^
**Age at first drink (years)** ^c^			
< 14	170 (23.94)	129 (26.12)	3.32 (1.75–6.29)^*^
≥14	578 (76.06)	19 (10.42)	Ref

a Among those who had ever used drugs using marijuana, amphetamines, cocaine, inhalants, cocaine, crack, ecstasy, glue, and ganja.

b Among those who had ever used cigarette.

c Among those who had ever drunk.

* *P*-value < 0.05.

### The associated risky sex behaviors with marijuana use

Table 4 presents the association between risky sex behaviors and various substance use. The odds of marijuana use were four times more likely among adolescents who have ever had sex compared with those who have not. Adolescents who engaged in sexual activity before the age of 14 years were approximately twofold more likely to have recently used marijuana than those who initiated after 14 years. No association was observed between the number of sexual partners and condom use in the last sex with marijuana use.

**Table 4:** Risky sex behaviors and their association with marijuana use among Jamaican adolescents in 7th−12th grades.

	**N (weighted %)**	**N (weighted %)**	**OR (95%CI)**
**Ever had sex**			
No	858 (51.91)	39 (4.65)	Ref
Yes	677 (48.09)	129 (20.75)	4.54 (2.55–8.06)^*^
**Age at first sex** ^a^			
< 14	370 (57.43)	39 (14.52)	2.45 (1.60–3.75)^*^
≥14	251 (42.57)	84 (26.55)	Ref
**Sexual partnership** ^a^			
Single partner	200 (30.72)	44 (25.37)	Ref
Multiple partners	418 (69.28)	79 (19.65)	0.74 (0.43–1.26)
**Condom use in last sex** ^a^			
No	389 (64.71)	76 (20.38)	1.30 (0.81–2.07)
Yes	195 (35.29)	41 (23.36)	Ref

a Among those who had ever sex.

* *P*-value < 0.05.

## Discussion

Our study revealed notable findings exploring the demographic factors associated with marijuana use among Jamaican adolescents from 7th to 12th grades. First, age appeared to be a significant predictor of recent marijuana use, with adolescents older than 14 years showing a substantially higher prevalence than those of 14 years and younger. This is in line with the findings from a study conducted by van den Bree, M (11) who noted that marijuana use typically began during the mid-adolescent years, particularly when peer influence and experimentation become more prominent. Additionally, sex differences in marijuana use were evident, with men showing a considerably higher prevalence than women. A similar sex pattern has been observed in several studies in various settings (12–14). According to this, societal norms and sex roles may play important roles in the observed differences. Interestingly, while our study highlighted that adolescents of 10th grade had the highest prevalence of marijuana use, this did not statistically differ from adolescents of 7th grade. However, adolescents of 12th grade had the lowest prevalence, contrary to the assumption that older adolescents may have increased exposure and opportunity to use substances such as marijuana. This discrepancy might be due to heightened awareness or preventive measures introduced in schools.

Our findings shed light on the multifaceted psychosocial risks and protective factors that are associated with marijuana use among Jamaican adolescents. The significant positive association between feelings of loneliness and increased marijuana use is congruent with the findings of research in other settings (15–17). For instance, a study by Savolainen et al. discovered that feelings of loneliness among adolescents were strongly linked to increased substance use, indicating a global trend (18). Similarly, the pronounced association between persistent worry and suicidal behaviors with marijuana use reflects the findings from other studies. These studies have consistently highlighted the connection between emotional distress and substance use, highlighting the idea that adolescents might use substances such as marijuana as a coping mechanism (19–21). An alarming revelation from our study is the increased marijuana use among adolescents who had been physically attacked, which highlights the need to investigate the complex interplay between trauma, substance use, and mental health among this population (22). Interestingly, the parental dimension in this study yielded striking insights. The findings indicated that adolescents with parents who smoke have increased odds of using marijuana, likely due to the tendency of children to emulate parental behaviors, especially those related to substance use (23). However, the protective role of parental support and awareness in deterring marijuana use cannot be understated. It reaffirms the power of a strong parental bond in shielding adolescents from potential harms (24). Based on these findings, policy recommendations might focus on creating more supportive environments for adolescents. Schools and communities could implement programs, such as emphasizing emotional wellbeing and ensuring adolescents to have avenues to discuss and express their feelings of loneliness, worry, or suicidal thoughts. Importantly, initiatives aiming to bolster parental awareness and support should be prioritized, given their pivotal role in mitigating marijuana use.

In our study, we observed several significant associations between various substance use behaviors and recent marijuana use among Jamaican adolescents from 7th to 12th grades. Notably, amphetamine use was found to have the strongest correlation with an adjusted odds ratio (AOR) of 24.49. This echoes the findings of previous research, indicating that polydrug use is prevalent among adolescents and that the co-use of substances can lead to increased risks of problematic behaviors and health outcomes (25). Furthermore, this study highlights the interconnectedness of substance use behaviors; the strong association between the use of current cigarette and other tobacco products and marijuana consumption is congruent with the previous research, suggesting that marijuana and tobacco often serve as gateway substances to one another (26). Alcohol consumption and early initiation of substance use (before the age of 14 years) were also identified as significant correlates. Adolescents with a history of getting drunk or troublingly intoxicated showed pronounced odds of recent marijuana use. Early substance initiation has been consistently linked with a higher risk of developing substance use disorders later in life (27, 28). Based on these findings, it is evident that early interventions targeting substance use education and prevention should be a priority. Policymakers might consider comprehensive substance use prevention programs that address a broad spectrum of substances rather than isolating marijuana. Additionally, future research could explore the underlying sociocultural and psychological factors contributing to these patterns among Jamaican adolescents, allowing for more tailored interventions.

With regard to risky sex behaviors and marijuana use among Jamaican adolescents, adolescents who had ever in sexual activity were more than four times as likely to have recently used marijuana compared with their counterparts who had not engaged in such behaviors (AOR = 4.54). Additionally, adolescents initiating sexual activity before the age of 14 years demonstrated higher propensity (AOR = 2.45) toward recent marijuana use compared with those starting after the age of 14 years. This trend is similar to previous studies, which indicated that early sexual debut correlates with elevated marijuana use (29, 30). Our study revealed no significant relationship between adolescents with multiple partners or those abstaining from condom use in their last sexual encounter and recent marijuana use, contributing to the complex interplay of sexual behaviors and substance use (29, 31, 32). Given the observed association between early sexual initiation and marijuana use, policymakers should consider joint interventions that address both early sexual debut and substance use among adolescents. Education programs in schools could be implemented, highlighting the implications of early sexual activity and the potential risks associated with substance use, particularly marijuana.

The results of the present study should be interpreted in light of several limitations. First, the data primarily come from secondary school students in Jamaica, potentially limiting the generalizability of our findings to broader age groups or different cultural contexts. The association between sex and the age of initiation of cannabis was notably significant in this demographic but might not be pronounced in other populations. Second, while we found a relationship between adolescents' perception of the harms and benefits of marijuana and their actual usage, the study did not delve deeper into the reasons for these perceptions, leaving potential motivational factors unexplored. Moreover, the data were drawn from the National Secondary School Survey of 2017, and given the rapidly evolving landscape of cannabis perception and legalization, the data might not be reflective of the current scenario. Finally, the survey indicated that a significant portion of adolescents of 8th grade did not perceive occasional cannabis use as harmful; however, the parameters for “occasional use” and “regular use” were not clearly defined, leading to potential ambiguities in the interpretation of these findings.

## Conclusion

This study delves into the multifaceted factors associated with marijuana use among Jamaican adolescents of 7th to 12th grades. The key findings highlight age and sex as significant determinants, with emotional distress markers such as feelings of loneliness, persistent worry, and suicidal behaviors having a notable positive correlation with marijuana use. Additionally, traumatic experiences and parental influence, both positive and negative, play pivotal roles in marijuana consumption patterns of adolescents. Furthermore, the intertwined relationship between early sexual debut, other substance use behaviors, and marijuana use highlights the need for comprehensive interventions that address a broad spectrum of substances and behaviors. To effectively mitigate marijuana use, policies should prioritize emotional wellbeing, parental support, early substance use education, and awareness of the implications of early sexual activity.

## Data availability statement

The original contributions presented in the study are included in the article/supplementary material, further inquiries can be directed to the corresponding author.

## Author contributions

OD: Conceptualization, Data curation, Formal analysis, Investigation, Methodology, Resources, Software, Writing – original draft, Writing – review & editing.
